# ZnWO_4_ Nanoparticle Scintillators for High Resolution X-ray Imaging

**DOI:** 10.3390/nano10091721

**Published:** 2020-08-31

**Authors:** Heon Yong Jeong, Hyung San Lim, Ju Hyuk Lee, Jun Heo, Hyun Nam Kim, Sung Oh Cho

**Affiliations:** Department of Nuclear and Quantum Engineering, Korea Advanced Institute of Science and Technology (KAIST), Daejeon 34141, Korea; jeong93@kaist.ac.kr (H.Y.J.); samsterdam@kaist.ac.kr (H.S.L.); aragorn477@kaist.ac.kr (J.H.L.); heojun@kaist.ac.kr (J.H.); trexpp@kaist.ac.kr (H.N.K.)

**Keywords:** nanoparticle, scintillator, zinc tungstate, X-ray, spatial resolution

## Abstract

The effect of scintillator particle size on high-resolution X-ray imaging was studied using zinc tungstate (ZnWO_4_) particles. The ZnWO_4_ particles were fabricated through a solid-state reaction between zinc oxide and tungsten oxide at various temperatures, producing particles with average sizes of 176.4 nm, 626.7 nm, and 2.127 μm; the zinc oxide and tungsten oxide were created using anodization. The spatial resolutions of high-resolution X-ray images, obtained from utilizing the fabricated particles, were determined: particles with the average size of 176.4 nm produced the highest spatial resolution. The results demonstrate that high spatial resolution can be obtained from ZnWO_4_ nanoparticle scintillators that minimize optical diffusion by having a particle size that is smaller than the emission wavelength.

## 1. Introduction

Digital X-ray imaging utilizes a converter which produces visible light (indirect) or electrons (direct) after absorbing X-rays. An indirect setup uses a scintillator as a converter coupled with a light detector [1,2,3,4]. A scintillator is a substance that emits visible light when exposed to X-rays or other forms of radiation. Powder scintillators are used ubiquitously in various industries, especially in those involving medical diagnostics and non-destructive testing [5,6,7,8,9]. Currently, many efforts are being made to identify small components within objects utilizing non-destructive testing with X-rays [10,11,12]. The general approach to achieving this is to use micro-focus X-ray tubes [10], which are, unfortunately, expensive and bulky. Another way of managing this is to utilize a high-resolution X-ray imaging system comprising a scintillator and a detector with a pixel size as small as several hundred nanometers when coupled with an optical lens [11,12]. The system utilizes X-rays to generate images with the scintillator positioned directly under the object of interest. The X-ray images generated by the scintillator are magnified by the optical lens.

Producing high spatial resolution is one of the most defining characteristics of high-resolution X-ray imaging [13]. Spatial resolution is affected by the structure of a scintillator [14,15,16]; thus, powder scintillators are commonly used for high-resolution X-ray imaging [13] as they are easy to handle and manipulate [11,17]. However, currently available powder scintillators are composed of particles that are micrometers in size [18,19], leading to optical diffusion and, ultimately, reduction in spatial resolution when the scintillators are applied to produce X-ray images [20,21,22].

To address this issue, there have been past studies to increase the spatial resolution of X-ray images by implementing nanoparticle scintillators [18,23]; the relatively small particle size mitigates optical diffusion. Liaparinos, P.F. analyzed the effect of scintillator grain size ranging from 1 to 1000 nm using Monte Carlo simulation based on Mie scattering theory [18]. The results from this paper showed that low optical diffusion can be achieved with nanoparticles. Cha, B.K., et al. produced europium-doped Gadolinium oxide (Gd_2_O_3_:Eu) nanoparticles and applied them to high-resolution X-ray imaging [23]. However, the effective pixel size of the detector used in the paper was far too large at 43 μm, and the study had issues with particle aggregation, leading to experimental failure.

In this study, zinc tungstate (ZnWO_4_) particles of various sizes were used to determine the effect of particle size on high-resolution X-ray imaging. ZnWO_4_ is a material of great interest in regard to being used as ultraviolet, X-ray, γ-ray, electron beam and proton beam scintillators [15,16,17,18,19], as the material is of low price and possesses excellent luminescence property, high density, high chemical stability, short decay time, and low afterglow [24,25,26,27,28,29,30,31]. The zinc oxide and tungsten oxide nanoparticles, used for fabricating ZnWO_4_, were synthesized using anodization. Subsequently, ZnWO_4_ nanoparticles were fabricated through a solid-state reaction between the as-prepared zinc oxide and tungsten oxide nanoparticles. The solid-state reaction was carried out at various temperatures to produce ZnWO_4_ particles of varying sizes. A thin layer of the particles was applied onto silicon substrates to be used as scintillators in high-resolution X-ray imaging, and the effective pixel size of the detector used in this study is less than 1 μm.

## 2. Materials and Methods

### 2.1. Materials

Zinc and tungsten wires were purchased from Goodfellow (Huntingdon, UK). Potassium chloride (KCl) powder, ammonium fluoride (NH_4_F) powder and polyethylene glycol (PEG, average molecular wight 200) were purchased from Sigma-Aldrich (St. Louis, MO, USA).

### 2.2. Fabrication and Characterization of ZnWO_4_ Particles

Voltages of 15 V and 80 V were applied to deionized (DI) water electrolyte solutions containing 1 M KCl and 1 M NH_4_F, respectively, to produce zinc oxide and tungsten oxide nanoparticles, respectively, via anodization. Anodization involves applying a voltage to a platinum electrode (−) and a metal wire (+) in an electrolyte solution, which leads to the oxidation and etching of the metal wire, resulting in the formation of metal oxide nanoparticles [32]. The nanoparticles were then extracted, washed with DI water, and dried in air at 60 °C. Afterwards, appropriate amounts of zinc oxide and tungsten oxide nanoparticles were added to DI water containing 0.2 wt% PEG to create solutions with a 1:1 atomic ratio between zinc and tungsten. The solutions were thoroughly mixed through sonication and vibrating ball milling, and then dried in air at 60 °C. The resulting powders were heated in an electric furnace at 700, 800, and 900 °C for 1 h to induce the solid-state reaction.

The structures of zinc oxide and tungsten oxide nanoparticles were analyzed using a field emission scanning electron microscope (FE-SEM, FEI MAGELLAN 400, FEI, Hillsboro, OR, USA). Further analyses were conducted using a transmission electron microscope (TEM, Titan cubed G2 60-300, FEI, Hillsboro, OR, USA) equipped with an energy dispersive X-ray spectroscopy (EDX) detector system (4 SDD Super X-detector system, FEI, Hillsboro, OR, USA). The structures of the fabricated ZnWO_4_ particles were also analyzed with a FE-SEM. The crystallinity of the ZnWO_4_ particles was analyzed using a high-resolution X-ray diffraction spectrometer (HR-XRD, SmartLab, Rigaku, Tokyo, Japan) with an analysis 2θ angle range of 10–90°.

### 2.3. Fabrication of ZnWO_4_ Scintillator

The fabricated ZnWO_4_ particles were mixed in DI water containing 0.2 wt% PEG and the mixture was ball-milled for 1 h to produce ZnWO_4_ paste. Subsequently, the ZnWO_4_ scintillators were developed by drop-casting the fabricated ZnWO_4_ particles onto 150 μm silicon glass substrates (1.5 cm × 1.5 cm) to a thickness of 10 μm.

## 3. Results and Discussion

### 3.1. Analyses of Nanoparticles Synthesized by Anodization

**Figure 1:**
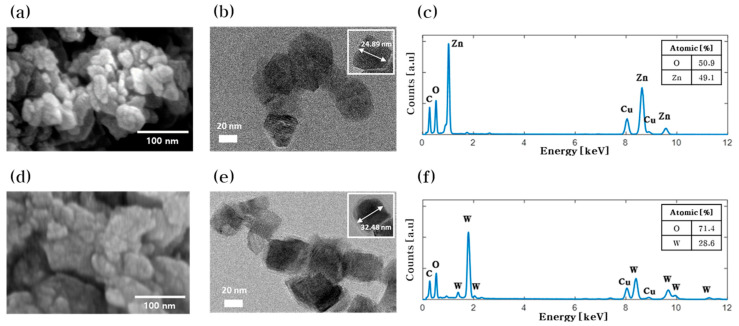
Metal oxide nanoparticles synthesized by anodization. (**a**) FE-SEM image, (**b**) TEM image, and (**c**) EDX spectrum of zinc oxide nanoparticles. (**d**) FE-SEM image, (**e**) TEM image, and (**f)** EDX spectrum of tungsten oxide nanoparticles.

The zinc oxide and tungsten oxide nanoparticles were prepared by anodization. Figure 1a,b are the images of zinc oxide nanoparticles acquired using the FE-SEM and TEM, respectively. The images show that the nanoparticles are spherical with a size of 20–40 nm. The FE-SEM and TEM images of tungsten oxide nanoparticles are shown in Figure 1d,e respectively. The images show that tungsten oxide nanoparticles are in the shape of a plate with a width of 20–60 nm. Figure 1c,f show EDX spectra obtained from the zinc oxide and tungsten oxide nanoparticles, respectively. Both EDX spectra have an oxygen peak, confirming that the nanoparticles were indeed oxidized; the observed copper and carbon peaks are from the use of TEM grids (carbon film on 300-mesh copper).

### 3.2. Structure of ZnWO_4_ Scintillator

**Figure 2:**
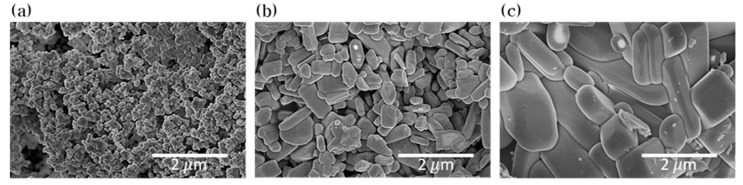
FE-SEM images of ZnWO_4_ fabricated at (**a**) 700 °C, (**b**) 800 °C, and (**c**) 900 °C.

**Figure 3:**
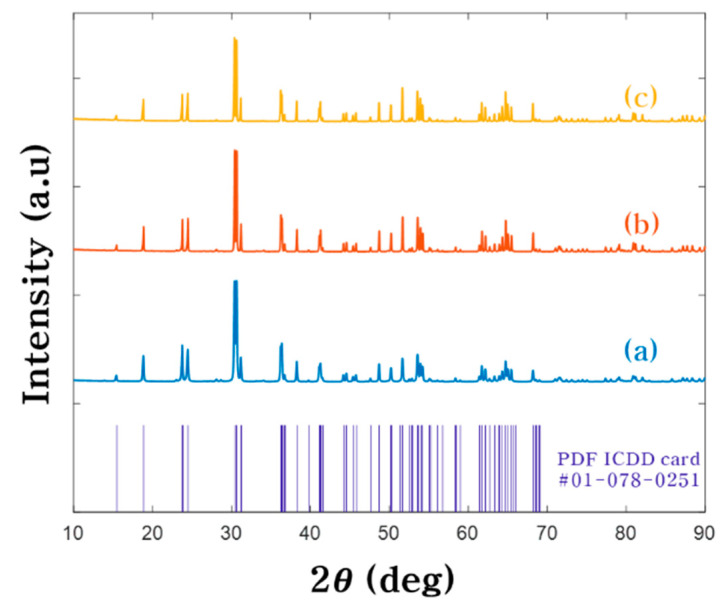
XRD spectra of ZnWO_4_ fabricated at (**a**) 700 °C, (**b**) 800 °C, and (**c**) 900 °C.

The ZnWO_4_ powders were fabricated by solid-state reaction at different temperature at 700, 800 and 900 °C. The structure morphology of ZnWO_4_ powders are showed in Figure 2 by FE-SEM. The ZnWO_4_ powders were sized through FE-SEM. As a result, the average sizes of powders fabricated at 700, 800 and 900 °C were 176.4 nm, 626.7 nm and 2.127 μm respectively. This phenomenon of particle size increase resulting from reaction temperature elevation can be explained by oriented attachment growth during the solid-state reaction [33]. From a thermodynamics viewpoint, the driving force behind oriented attachment growth is reduction in free surface energy after particle mergence [34,35]. The ZnWO_4_ powders fabricated at 700 and 800 °C were formed in the wavelength band of light or smaller, and the size of the powders fabricated at 900 °C were formed to micro-sized particles larger than the wavelength of light. Figure 3 shows the XRD patterns of the ZnWO_4_ powders with an analysis 2θ range 10–90 °C. Comparing the peak value of the XRD pattern with the standard card (PDF ICDD card #01-078-0251), these powders crystallized into pure monoclinic ZnWO_4_.

### 3.3. Evaluation of X-ray Iimaging Performance in High-resolution X-ray Imaging

**Figure 4:**
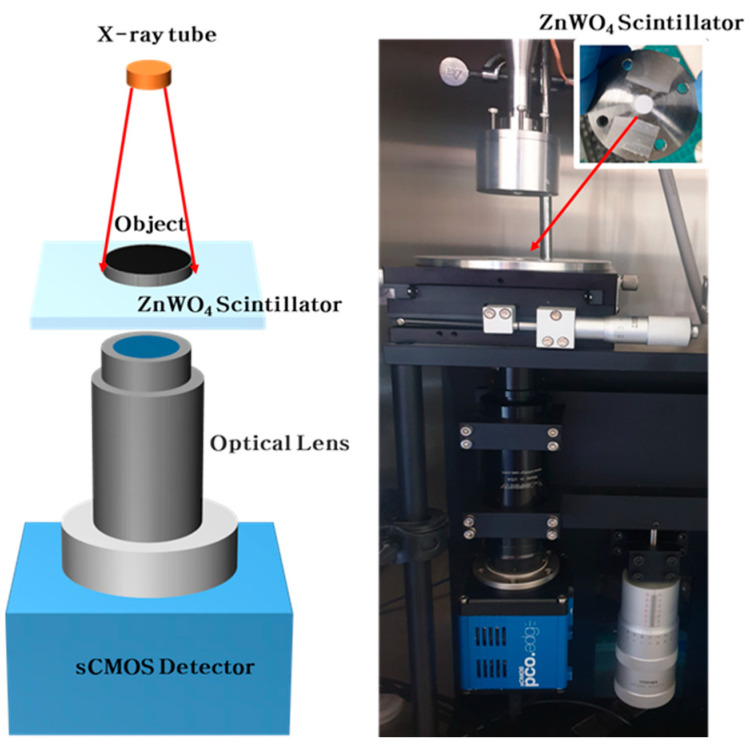
Applying ZnWO_4_ scintillator screen to high-resolution X-ray imaging system.

The fabricated scintillator screens were implemented into a high-resolution X-ray imaging system (Figure 4). This system consists of a carbon nanotube (CNT) based miniature X-ray tube, a scintillator, an optical lens, and a scientific complementary metal-oxide-semiconductors (sCMOS) detector. The X-ray tube utilizes a CNT based electron beam emitter with an operating voltage and current of 50 kV_p_ and 0.35 mA, respectively. The focusing lens, attached to the X-ray tube, is cylindrical with an outer diameter of 40 mm and a height of 9 mm and is comprised of permanent magnets (NdFeB). The focal spot size of the miniature X-ray tube with the focusing lens is 337–390 μm [26]. A 10x optical lens is used to magnify the images produced by the scintillator and a sCMOS detector is used to develop the images. The pixel size of the sCMOS detector is 6.5 μm, but when coupled with the optical lens, the effective pixel size is reduced to 0.65 μm.

**Figure 5:**
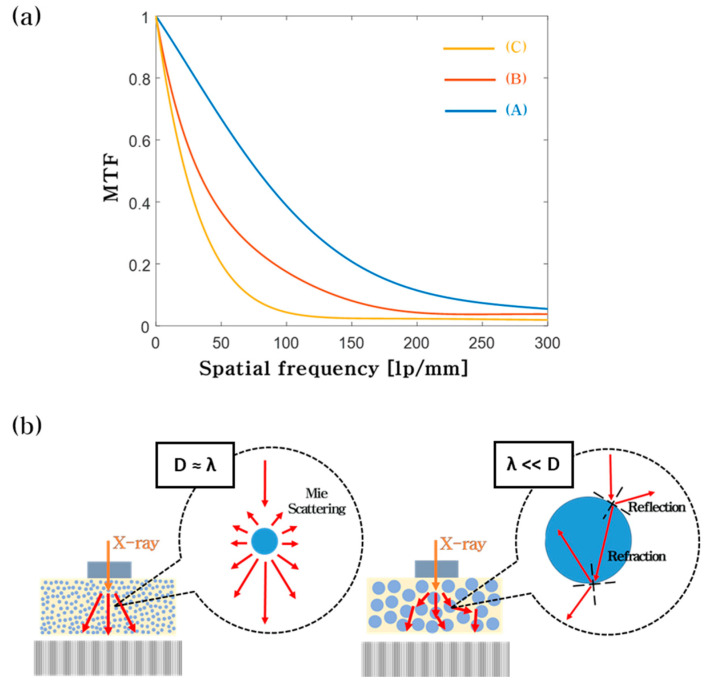
(**a**) MTF curves obtained from ZnWO_4_ particles with sizes of (A) 176.4 nm, (B) 626.7 nm, and (C) 2.127 μm. (**b**) The effect of scintillator size on the spatial resolution of an X-ray image.

The relative emission intensities from the three fabricated ZnWO_4_ scintillators, each with a different particle size, were measured using the high-resolution X-ray imaging system. The emission intensity values were determined by averaging the pixel values of X-ray images obtained at the same exposure condition. The results indicate no significant differences in emission level between the ZnWO_4_ scintillators (Figure S1). The modulation transfer functions (MTFs) were measured to evaluate the spatial resolutions for the fabricated ZnWO_4_ particles. This procedure is highly common in the field of X-ray imaging [12,23,36,37,38]. X-ray images of an edge of a 0.5 cm thick tungsten block were developed using the high-resolution X-ray imaging fitted with the fabricated ZnWO_4_ scintillator screens. The edge spread functions (ESFs) were determined from the obtained X-ray images and, in turn, the MTFs were determined from the ESFs. The MTF curves obtained from utilizing the different scintillator screens are shown in Figure 5a. The spatial frequency values of 278.2, 204.1, and 124.1 lp/mm were achieved from the average particle sizes of 176.4 nm, 626.7 nm, and 2.127 μm, respectively, at the MTF value of 10%.

The reduction in spatial frequency resulting from particle size increase can be explained by the mechanism behind optical diffusion. For particles with a size much greater than the emission wavelength (λ), geometrical optics apply [39,40]. With such particles, light is reflected and refracted at the particle surface, causing significant light scatter. This is the case for the ZnWO_4_ particles with an average size of 2.127 μm, resulting in the application of the particles producing the lowest frequency among the frequency values obtained. When the particle size is comparable to the emission wavelength, Mie scattering becomes relevant [41,42]. Mie scattering is a phenomenon that causes the probability of light scatter to decrease steeply with decreasing particle size [43,44,45]. The ZnWO_4_ emission spectrum covers a wavelength range of 400–600 nm when excited by X-rays at room temperature [24]. To verify, the photoluminescence (PL) spectra of the ZnWO_4_ particles of different sizes were measured with a PL spectrometer (RAMBOSS-Star, DONGWOO OPTRON, Gyeonggi-do, Korea) equipped with a UV laser (325 nm He-Cd Laser, Kimmon, Tokyo, Japan). All spectra were close to identical and roughly covered a wavelength range of 400–600 nm (Figure S2). Thus, it can be expected that the 176.4 nm particles exhibit the highest spatial frequency.

**Figure 6:**
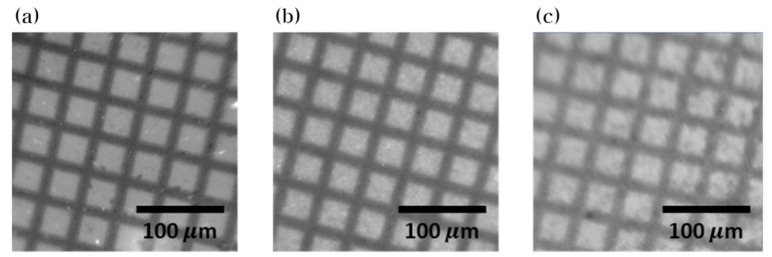
X-ray images of a TEM grid obtained from utilizing ZnWO_4_ scintillator screens with average particles sizes of (**a**) 176.4 nm, (**b**) 626.7 nm, and (**c**) 2.127 μm.

Figure 6 shows the X-ray images of 300-mesh copper TEM grid obtained from the high-resolution X-ray imaging system. Consistent with the MTF results, a smaller particle size leads to a higher image resolution. The small effective pixel size (0.65 μm) allows for the inspection of small objects, however, causes the system to be sensitive to light scatter. Thus, it can be concluded that the use of small nanoparticles is optimal in improving the spatial resolution of X-ray images.

## 4. Conclusions

The ZnWO_4_ scintillator particles were fabricated through anodization and a solid-state reaction. The fabricated particles were then applied to high-resolution X-ray imaging to evaluate the spatial resolutions of the produced X-ray images. The experimental results showed that the optimal spatial resolution is achieved when the average particle size is less than the emission wavelength. The ZnWO_4_ nanoparticles (100–250 nm), developed in our study, can be expected to reduce optical diffusion and provide spatial resolution. Furthermore, the significance of our results is not limited to ZnWO_4_ particles; it can also be applied to other scintillator particles.

## Supplementary Materials

The following are available online at https://www.mdpi.com/2079-4991/10/9/1721/s1, Figure S1: The emission intensities of ZnWO_4_ scintillator screens with average particles sizes of (a) 176.4 nm (700 °C), (b) 626.7 nm (800 °C), and (c) 2.127 μm (900 °C); Figure S2: PL spectra of ZnWO_4_ particles with average particles sizes of (a) 176.4 nm, (b) 626.7 nm, and (c) 2.127 μm.
