# Supplementary material for: ZnWO4 Nanoparticle Scintillators for High Resolution X-ray Imaging
Source: Nanomaterials (Basel). 2020 Aug 31;10(9):1721. doi: 10.3390/nano10091721 (PMC7559253; doi:10.3390/nano10091721)
Supplement: Supplementary file 1 [file nanomaterials-10-01721-s001.pdf]

*nanomaterials*

MDPI

*Article*

# $ZnWO_4$ Nanoparticle Scintillators for High-Resolution X-ray Imaging

**Heon Yong Jeong, Hyung San Lim, Ju Hyuk Lee, Jun Heo, Hyun Nam Kim and Sung Oh Cho ***

Department of Nuclear and Quantum Engineering, Korea Advanced Institute of Science and Technology (KAIST), Daejeon 34141, Korea; jeong93@kaist.ac.kr (H.Y.J.); samsterdam@kaist.ac.kr (H.S.L.); aragorn477@kaist.ac.kr (J.H.L.); heojun@kaist.ac.kr (J.H); trexpp@kaist.ac.kr (H.N.K.);

* Correspondence: socho@kaist.ac.kr; Tel.: +82-(0)42-350-3823; Fax: +82-(0)42-350-3810

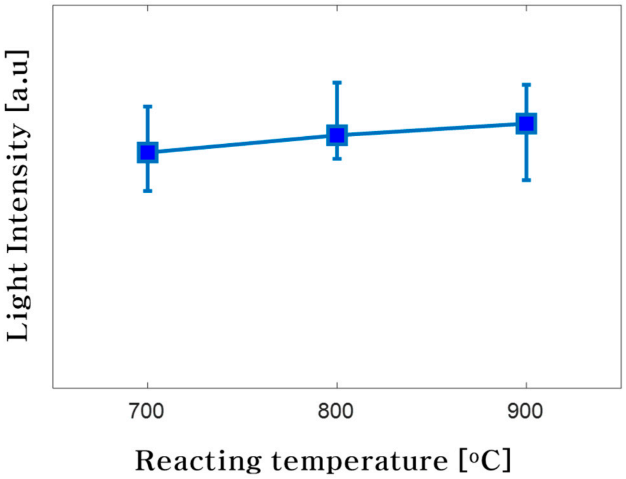


**Figure 1.** The emission intensities of $ZnWO_4$ scintillator screens with average particles sizes of (a) 176.4 nm (700°C), (b) 626.7 nm (800°C), and (c) 2.127 µm (900°C).

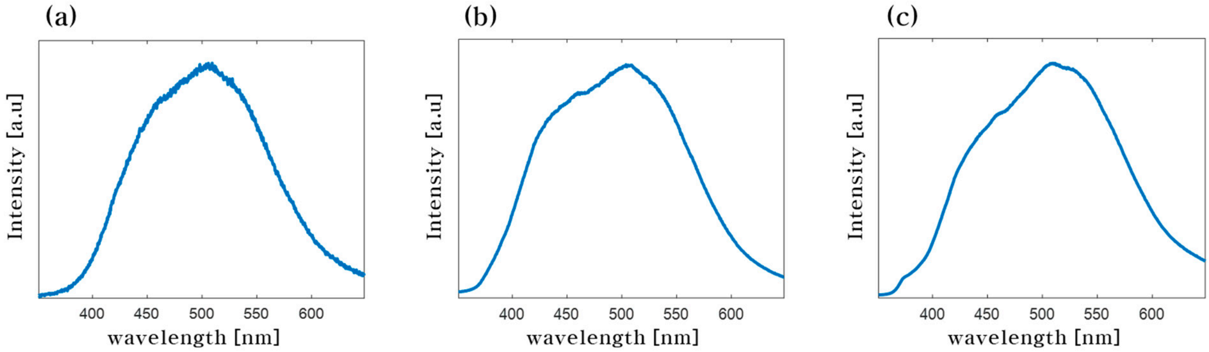


**Figure 2.** PL spectra of $ZnWO_4$ particles with average particles sizes of (a) 176.4 nm, (b) 626.7 nm, and (c) 2.127 µm.
